# Mobile Recommendation Based on Link Community Detection

**DOI:** 10.1155/2014/259156

**Published:** 2014-08-26

**Authors:** Kun Deng, Jianpei Zhang, Jing Yang

**Affiliations:** College of Computer Science and Technology, Harbin Engineering University, Harbin 150001, China

## Abstract

Since traditional mobile recommendation systems have difficulty in acquiring complete and accurate user information in mobile networks, the accuracy of recommendation is not high. In order to solve this problem, this paper proposes a novel mobile recommendation algorithm based on link community detection (MRLD). MRLD executes link label diffusion algorithm and maximal extended modularity (EQ) of greedy search to obtain the link community structure, and overlapping nodes belonging analysis (ONBA) is adopted to adjust the overlapping nodes in order to get the more accurate community structure. MRLD is tested on both synthetic and real-world networks, and the experimental results show that our approach is valid and feasible.

## 1. Introduction

Along with the rapid development of the mobile communication technology, mobile devices have gradually become one of the most important platforms for people to acquire information. Compared with the traditional internet PC terminal, mobile devices have a lot of advantages such as strong mobility, good portability, and easy access, which allow people to get service and information on mobile networks at any time. Meanwhile, as the information in mobile networks is increasing constantly, the amount of information is now beyond the acceptability of the people. Moreover, the performances of mobile devices in terms of interface display, terminal processing, as well as input and output are still limited and have led to serious information overload issues for people [1]; thus, the utilization ratio of network resources is severely influenced. Therefore, how to identify the information which attracts the users most from vast amounts of information has become an urgent issue in mobile networks personalized service.

In recent years, personalized information recommendation service provided by mobile recommendation systems has become an effective solution to mobile information overload issues. However, in consideration of their privacy and security, mobile users are reluctant to provide precise and complete information to the mobile recommendation systems, which influenced the accuracy of the mobile recommendation systems greatly.

Currently, the community detection technology in complex networks provides powerful support to solve the above problems. The reason for this is that the community structure in complex networks often possess the feature of “dense intracommunity connections and sparse intercommunity connections” [2], in which intracommunity usually represents a group of people who share some similar characteristics; for example, in mobile networks, people often form their own mobile communication communities according to their professions and interest. Because of the same background and interest, the users in the same community are likely to have the same information demands (e.g., the same kind of movie, music, news, etc.). Therefore, the utilization of community detection technology could divide all mobile users into different communities, and then according to the property of every community that the users usually have the same information demands, it could provide accurate information service for the whole community with only a small part of user information.

To sum up, excellent community detection method is essential in completing a recommendation task accurately by community detection technology in mobile networks. However, traditional community detection method is to divide the network into some disconnect communities, and every node belongs to only one community, while in mobile networks, communities are incomplete independent and are overlapped, which means some nodes could be members of different communities at the same time [3]. Therefore, overlapping community detection in mobile networks is usually of more practical significance. Although previous researches on community detection mainly focused on identifying the communities of nodes, studies have shown that [4] link community detection usually has more advantages in detecting overlapping communities, that is, because a node may carry multiple identities, which means it belongs to multiple communities at the same time. For instance, a person may belong to multiple communities at the same time, such as families, friends, and coworkers. In contrast, a link in a network usually indicates the unique relationship between a pair of nodes; it has one single role and belongs to only one community. When all the communities are determined, the corresponding overlapping nodes will naturally belong to multiple communities. Therefore, link community has obvious advantage in detecting overlapping communities and provides powerful technical support for the improvement of the accuracy of mobile recommendation.

Based on the above analysis, an algorithm is proposed in this paper, called mobile recommendation based on link community detection (MRLD), which could perform accurate group recommendation to mobile users without having to get all the user information in mobile networks. MRLD algorithm first initializes the label of every link to the node label which possesses the higher degree when connected by the link and gets the links according to the degree of the nodes (the nodes are connected by the links) in descending order. Then executes triangle label diffusion to aggregate the links with the same label to one community, thus, to form an original community structure with dense intracommunity connection; secondly, we combine the obtained communities with maximal extended modularity (EQ) [5] of greedy search to obtain the optimal overlapping community structure; thirdly, overlapping nodes belonging analysis (ONBA) is adopted to adjust the overlapping nodes to avoid “excessive overlapping” which had happened in link community detection; finally, on the basis that overlapping communities are detected precisely, accurate information targeted on every community in mobile networks is recommended to the users.

The rest of this paper is organized as follows.  Section 2 surveys the related work.  Section 3 explains and justifies the design of our algorithm, named MRLD. In Section 4, we discuss the experiments of MRLD algorithm.  Section 5 concludes this paper.

## 2. Related Works

### 2.1. Mobile Networks Recommendation

As mobile devices could approach users much easier than traditional internet PC terminals, mobile users are likely to have more realistic social behaviors in mobile networks; therefore, many recommendation approaches based on social relationships in mobile networks are proposed. For example, reference [6] built a collaborative filtering algorithm based on the friend relations of social websites and accomplished the collaborative recommendation task, and the experimental results suggested that the friend relations in social websites could improve the accuracy of collaborative filtering algorithm; reference [7] decomposed the obtained score matrix of the users and discovered the potential factors to influence the scores, meanwhile, it took the influence of the trust among friends into consideration and accomplished the group recommendation; reference [8] combined collaborative filtering algorithm with social trust networks and discovered the neighbors of the users through the score of social trust networks, thus, recommendation is well performed by collaborative filtering algorithm; reference [9] obtained the mobile networks in which users interact with each other by the frequency and duration of the interaction, in other words, it used the frequency and duration of the interaction to figure out the strength ties between the users, meanwhile, it analyzed the distance (the distance refers to the sum of the link weights of the pathway between any two nodes, and the weights of links represent the strength of the users' relationship) between two nodes to estimate the possibility of two nodes being friends. Finally, the nodes that have stronger connection strength with a user are recommended to the user as potential friends.

### 2.2. Community Detection

Recently, various kinds of community detection algorithms have been proposed, for example, GN algorithm [10], proposed by Girvan and Newman, considers that the edge betweenness connected between the communities should be higher than the edge betweenness connected inside the community. The edge betweenness of an edge can be defined as “the number of the shortest paths in the network which pass through the edge and connect any two nodes.” GN algorithm obtained a dendrogram concerning community structure by deleting iteratively the edge with the biggest edge betweenness, and Newman and Girvan defined modularity (*Q*) function [2] to cut the dendrogram and obtained the optimal community structure. After that, optimization algorithm based on modularity function has become one of the main stream algorithms in community detection. However, these algorithms are to decompose the networks into several disconnected communities and every node must belong to only one community. But in real-world networks, communities are incomplete independent and are overlapped. As a result, many overlapping community detection algorithms have been proposed, for example, CPM algorithm based on clique percolation [3] considers that the edges inside the community are likely to form big complete subgraphs, while the edges between the communities are almost impossible to form big complete subgraphs; hence CPM algorithm uses clique percolation to detect the community structure; algorithms based on local optimization [11, 12] constantly expand the local community where different seeds are located by optimizing fitness functions, thus, the global optimal community structure is obtained; algorithms based on label propagation [13–15] first initialized a unique label with belonging coefficient for every node. Then they, repeatedly, modify the labels by summing and normalizing the belonging coefficients of the neighbor nodes which have the same labels. Finally, nodes with the same labels belong to the same community, and the overlapping nodes possess multiple labels.

Research has shown [4] that link community detection is usually more advantageous in detecting overlapping communities. Therefore, many link community detection approaches are proposed. For example, reference [16] converted the original network structure into line graph structure and then employed random walk method to detect the overlapping community structure; reference [4] proposed a hierarchy clustering algorithm based on link similarity. This algorithm first generated a dendrogram of link community structure, then it use partition density function *D* to cut the dendrogram, through which the final community structure is obtained; reference [17] extended the map equation method of node communities to link communities and modified the coding rule of random walk in order to complete link community detection.

This paper takes advantage of the superior capability of link community in detecting overlapping communities and proposes a community detection algorithm which could accomplish information recommendation task by precisely detecting overlapping communities in mobile networks.

## 3. The Algorithm

MRLD algorithm first adopts link label diffusion algorithm to create the original community structure; secondly, it combines the communities with maximal extended modularity (EQ) of greedy search to obtain the optimal overlapping community structure; thirdly, the algorithm analyzes and adjusts the obtained link community structure through overlapping nodes belonging analysis (ONBA), and then gets the ultimate overlapping community structure; finally, based on the acquired densely connected overlapping community structure, recommendation to all the users of the community could be implemented.

### 3.1. Link Label Diffusion Algorithm

This section takes links as research object and proposes an algorithm, named link label diffusion algorithm (LLDA), to acquire the original link community structure, and before the detail description of the algorithm, the following definitions and theorem should be provided first. Assume a social network *G* = (*V*, *E*), here *V* denotes the set of nodes and *E* denotes the set of edges (or links) of *G*.

**Definition 1 (neighbor link).**

Assume *l*_*v*,*v*′_ ∈ *E*, then the neighbor link *n*(*l*_*v*,*v*′_) of link *l*_*v*,*v*′_ could be defined as the set of links which connect node *v* or *v*′, and *l*_*v*,*v*′_ do not belongs to this set, that is: *n*(*l*_*v*,*v*′_) = {*l*_*v*,*v*′_ ∈ *E*∣*i* ∈ *n*(*v*) and *i* ≠ *v*′}∪{*l*_*v*,*v*′_ ∈ *E*∣*j* ∈ *n*(*v*′) and *j* ≠ *v*}, where *n*(*v*) and *n*(*v*′) represent the sets of the neighbor nodes of *v* and *v*′, respectively.

**Definition 2 (triangle).**

Assume *i*, *j*, *k* ∈ *V* and *i*, *j*, *k* are the three vertexes of triangle Δ*k*. Then triangle Δ*k* could be defined as any two of the three vertexes *i*, *j*, *k* are connected by links, that is: Δ*k* = {*i*, *j*, *k* ∈ *V*∣*l*_*i*,*j*_ ∈ *E*, *l*_*j*,*k*_ ∈ *E*, *l*_*i*,*k*_ ∈ *E*}.

**Definition 3 (partition density function, see [4]).**

Assume in community *C*_*i*_, *m*_*c*_*i*__ is the number of links and *n*_*c*_*i*__ is the number of nodes, then the partition density function is defined as the following formula:

(1) $$\begin{array}{c} D_{c_{i}}=\frac{m_{c_{i}}-\left(n_{c_{i}}-1\right)}{n_{c_{i}}\left(n_{c_{i}}-1\right)/2-\left(n_{c_{i}}-1\right)}, \end{array}$$

where when *n*_*c*_*i*__ = 2, assume *D*_*c*_*i*__ = 0. Formula (1) suggests that in *C*_*i*_, more links and fewer nodes indicate a denser intracommunity connection.

**Definition 4 (link label).**

Assume link *l*_*v*,*v*′_ belongs to community *C*_*i*_, and *L*(*C*_*i*_) is the community identifier of *C*_*i*_, if *L*(*l*_*v*,*v*′_) = *L*(*C*_*i*_), then *L*(*l*_*v*,*v*′_) is called the link community label of link *l*_*v*,*v*′_ (link label in abbreviation).

**Theorem 5 .**

Given nodes *i*, *j*, *k* ∈ *V*, links *l*_*i*,*j*_, *l*_*i*,*k*_, *l*_*j*,*k*_ ∈ *E*, *C*_*u*_ is a community, *m*_*u*_ is the number of links in *C*_*u*_, and *n*_*u*_ is the number of nodes, where *l*_*i*,*j*_, *l*_*i*,*k*_ ∈ *C*_*u*_, *l*_*j*,*k*_ ∉ *C*_*u*_, if join *l*_*j*,*k*_ in community *C*_*u*_ to constitute the community *C*_*u*′_, then the partition density of *C*_*u*′_ (denoted *D*_*c*_*u*′__) is equal or larger than the partition density of *C*_*u*_ (denoted *D*_*c*_*u*__).

**Proof**

The partition density *D*_*c*_*u*__ of community *C*_*u*_ can be defined as follows:

(2) $$\begin{array}{c} D_{c_{u}}=\frac{m_{u}-\left(n_{u}-1\right)}{n_{u}\left(n_{u}-1\right)/2-\left(n_{u}-1\right)}. \end{array}$$

If join *l*_*j*,*k*_ in *C*_*u*_ to constitute a community *C*_*u*′_, then the partition density *D*_*c*_*u*′__ of community *C*_*u*′_ can be defined as

(3) $$\begin{array}{c} D_{c_{u^{\prime }}}=\frac{m_{u^{\prime }}-\left(n_{u^{\prime }}-1\right)}{n_{u^{\prime }}\left(n_{u^{\prime }}-1\right)/2-\left(n_{u^{\prime }}-1\right)}. \end{array}$$

The number of links is added by one after the joining of *l*_*j*,*k*_ into community *C*_*u*_, so *m*_*u*′_ = *m*_*u*_ + 1. Because *l*_*i*,*j*_, *l*_*i*,*k*_ ∈ *C*_*u*_, nodes *i*, *j*, *k* is connected by *l*_*i*,*j*_ and *l*_*i*,*k*_, so nodes *i*, *j*, *k* are already members of community *C*_*u*_. That is to say, the joining of *l*_*j*,*k*_ into community *C*_*u*_ to form a new community *C*_*u*′_ would not change the number of nodes, that is, *n*_*u*′_ = *n*_*u*_, then formula (3) could also be defined as

(4) $$\begin{array}{c} D_{c_{u^{\prime }}}=\frac{\left(m_{u}+1\right)-\left(n_{u}-1\right)}{n_{u}\left(n_{u}-1\right)/2-\left(n_{u}-1\right)}. \end{array}$$

Therefore, *D*_*c*_*u*′__ > *D*_*c*_*u*__. This concludes this proof.

Theorem 5 shows that if two links of a triangle belong to the same community, then the joining of the third link would increase the partition density of the community. Based on this theorem, LLDA algorithm is proposed in this paper. Research has shown [18] that the node with higher degree has stronger influence in the local range, and it could absorb the surrounding nodes to form a community. So LLDA algorithm first initializes the label of every link to node label which possesses the higher degree when connected by the link, thus, to form an original community with tree structure. Then, in this network, get links *l*_*i*,*j*_ according to the degree of the nodes (the nodes are connected by the links) in descending order. Meanwhile, search triangle set Δ*l*_*i*,*j*_ which includes *l*_*i*,*j*_, if the other two links of triangle Δ*k* in set Δ*l*_*i*,*j*_ have the same label, then the label of *l*_*i*,*j*_ is modified to the label of the other two links. This process is also called triangle label diffusion. At this moment, if there is still link *l*_*a*,*b*_ which does not belong to any community, then the label of *l*_*a*,*b*_ is modified to the most frequent label of its neighbor links. After the above process, links with the same label are attributed to the same community. Based on this idea, LLDA algorithm could be expressed as shown in Algorithm 1.

**Figure 1:**
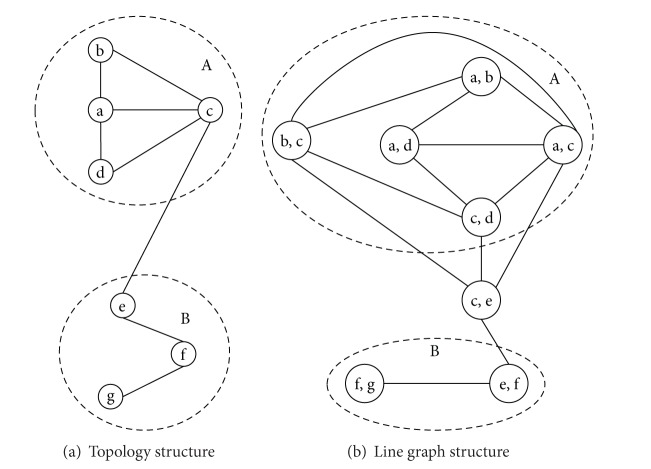
Network topology structure and line graph structure with 7 nodes.

In Algorithm 1, steps (1)–(3) are the initialization process of link labels, where *T*(*l*_*i*,*j*_) is the label modification marker of link *l*_*i*,*j*_, and its purpose is to make sure the label of every link could only be modified once after the initialization, thus, to ensure every link is in the same community with the node which possess the highest degree in the local range. Step (5) is to get links *l*_*i*,*j*_ according to the degree of the nodes (the nodes are connected by the links) in descending order. The aim is to make sure that in this diffusion process, link labels diffuse from the links with higher node degree to the links with lower node degree. Steps (6)–(16) are to perform triangle label diffusion to the labels of the links. According to Theorem 5, this operation is reasonable and a dense link community structure is obtained by the end of this process. By this time if there are still links with label modification marker *T*(*l*_*i*,*j*_) = 0, then it means that these links are not detected to any community. Steps (17)–(19) are followed to modify the labels of the links whose label modification marker *T*(*l*_*i*,*j*_) = 0 to the most frequent labels of their neighbor links. The fundamental of this process is that the number of the neighbor links which belong to the same community with a link could reflect the tendency of the link to a community, For example, Figure 1 (Figure 1(b) is the line graph structure correspond to the network topology structure of Figure 1(a)) shows that link *l*_*c*,*e*_ has a denser connection with community A, so it is more reasonable to attribute link *l*_*c*,*e*_ to community A.

From Algorithm 1 we can see that the time complexities of step (5) and steps (6)–(15) are the highest. Obviously, the time complexity of step (5) is *O*(*n* ^2^). Steps (6)–(15) are to execute triangle label diffusion to the *m* links in the networks, so,* labelList* multiplies* dnEdges* is equal to *m*. And because the number of triangles which include a specific link in the networks is *t*, therefore, the running time of steps (6)–(16) is *O*(*tm*). However, *t* is usually constant, so the time complexity of this operation is *O*(*m*). To sum up, the time complexity of LLDA is *O*(*m* + *n* ^2^).

After the execution of LLDA, links with the same label are aggregated to the same community, indicating the accomplishment of original community construction phase. This process have shown that link labels diffuse from the links with higher node degree to the links with lower node degree, thus, aggregated the densely connected links to one community in the local range.

### 3.2. The Combination of Link Communities

Though multiple communities with dense intraconnections are achieved after the execution of LLDA algorithm, the corresponding community structure may not be the global optimal structure, so it is still necessary to combine the link communities to achieve the global optimal community structure. This paper employed the maximal extended modularity (EQ) of greedy search to combine the obtained communities in order to achieve the optimal overlapping community structure.

**Definition 6 (extended modularity, EQ).**

Assume *m* is the number of links in the network, *C*_*i*_ is the *i*th community in the community structure, *O*_*v*_ is the number of belonging communities of node *v*, *k*_*v*_ is the degree of node *v*, and *A* is the corresponding adjacent matrix of the whole network, then the EQ can be defined as follows:

(5) $$\begin{array}{c} \text{EQ}=\frac{1}{2m}\underset{i}{\sum }\underset{u,v\in C_{i}}{\sum }\frac{1}{O_{u}O_{v}}\left[A_{u,v}-\frac{k_{u}k_{v}}{2m}\right]. \end{array}$$

Here, if nodes *u*, *v* is connected by a link, then *A*_*uv*_ = 1, otherwise *A*_*uv*_ = 0. The implication of EQ is that, a higher EQ value indicates a denser intracommunity structure, that is, a more qualified community structure.

According to the definition of EQ, the EQ value of the community structure is the accumulation of the EQ value of every community, if the combination of two communities increases the EQ value, then it means that the combined community structure is superior to the community structure before the combination. Hence we can do the following modification to the function of EQ, given two communities *c*_*x*_ and *c*_*y*_, then the functions of EQ_*c*_*x*__ and EQ_*c*_*y*__ can be defined as follows, respectively:

(6) $$\begin{array}{c} {\text{EQ}}_{c_{x}}=\frac{1}{2m}\underset{u,v\in c_{x}}{\sum }\frac{1}{O_{v}O_{u}}\left[A_{uv}-\frac{k_{u}k_{v}}{2m}\right],\\ {\text{EQ}}_{c_{y}}=\frac{1}{2m}\underset{u,v\in c_{y}}{\sum }\frac{1}{O_{v}O_{u}}\left[A_{uv}-\frac{k_{u}k_{v}}{2m}\right]. \end{array}$$

If add the EQ values of *c*_*x*_ and *c*_*y*_, then

(7) $$\begin{array}{c} {\text{EQ}}_{c_{x}+c_{y}}={\text{EQ}}_{c_{x}}+{\text{EQ}}_{c_{y}}. \end{array}$$

If the new community obtained after the combination of *c*_*x*_ and *c*_*y*_ is denoted by *c*_*z*_, then the EQ value of *c*_*z*_ can be demonstrated as the following formulation:

(8) $$\begin{array}{c} {\text{EQ}}_{c_{z}}=\frac{1}{2m}\underset{u,v\in c_{z}}{\sum }\frac{1}{O_{v}O_{u}}\left[A_{uv}-\frac{k_{u}k_{v}}{2m}\right]. \end{array}$$

At this moment, the increment of EQ_*c*_*z*__ to EQ_*c*_*x*_+*c*_*y*__ (denoted ΔEQ) can be defined as

(9) $$\begin{array}{c} \Delta \text{EQ}={\text{EQ}}_{{c_{z}}_{}}-{\text{EQ}}_{c_{x}+c_{y}}. \end{array}$$

According to the above analysis, if the value of ΔEQ is higher, it means that the combined community makes more contribution to the value of EQ. Based on this, the ideology of community combination is to compute the ΔEQ of every pair of adjacent communities, and then to combine the communities with the largest ΔEQ value. Iterate the above process until the ΔEQ of every pair of adjacent communities is less than or equal to zero. Thus, it can be seen that the final community structure after the iteration correspond the optimal EQ value.

It can be easily seen that, if LLDA algorithm divide the whole community into *s* original communities, and the average number of nodes in every community is *p*, then the time complexity of every combination is *O*(*p* ^2^). In the worst case, the algorithm would combine the communities into one community and the time complexity is *O*(*sp* ^2^). Since *p* is far lower than *n*, and *s* is constant, the time complexity of this phase is far below *O*(*n* ^2^).

### 3.3. Overlapping Nodes Belonging Analysis

**Figure 2:**
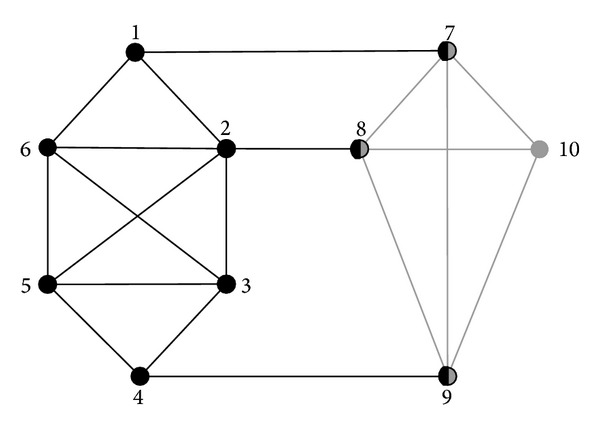
Instance of link community.

Since the detection of link communities is the detection of communities that the links belong to, so if a node is connected by multiple links and one of the links belongs to a different community, then this node is to be identified as an overlapping node. As is illustrated in Figure 2, nodes 7, 8, 9, 10 have already form a dense community, but each node of the nodes 7, 8, 9 is connected to another community by a link, so nodes 7, 8, 9 will be identified as overlapping nodes in the detection process. Therefore, we can come to the conclusion that in link community detection algorithms, nodes have higher probability of being identified as overlapping nodes, so the detected communities are likely to be “excessive overlapping” which decreased the accuracy of community detection.

In order to solve the problem of “excessive overlapping” in link community detection, this paper proposes overlapping nodes belonging analysis (ONBA). Before discussing ONBA in detail, we will introduce the definition of community belonging value.

**Definition 7 (community belonging value).**

Given link community structure *C* = {*C*_1_, *C*_2_,…, *C*_*k*_}, in which *C*_*i*_ represents any community in *C*, *k* is the number of communities in *C*, and *O* is the set of overlapping nodes, then the belonging value of any overlapping node *v* belongs to community *C*_*i*_ can be defined as follows:

(10) $$\begin{array}{c} \Gamma_{v,C_{i}}=\frac{k_{v,C_{i}}}{k_{v}}. \end{array}$$

Here, *k*_*v*,*C*_*i*__ is the number of links which connect node *v* with the nodes in *C*_*i*_, *k*_*v*_ is the degree of node *v*, and Γ_*v*,*C*_*i*__ is the belonging value of node *v* which belongs to community *C*_*i*_; then we can use Γ_*v*,*C*_*v*__ = {Γ_*v*,*C*_1__, Γ_*v*,*C*_2__,…, Γ_*v*,*C*_*t*__} to represent the set of belonging values that node *v* belongs to *C* = {*C*_1_, *C*_2_,…, *C*_*t*_}, and *t* is the number of the adjacent communities of node *v*.

Based on the definition of community belonging value, this paper proposes overlapping nodes belonging analysis (ONBA). The basic ideology of this analysis is: if a node connect densely with some communities, but connect sparsely with the others, then the overlapping node has the tendency of belonging to the communities which are densely connected with it, that is, the node belongs to the communities; if an overlapping node has no obvious tendency to any community, in other words, the number of links which connected the node with each of its adjacent communities have no obvious difference, then no adjustment is made to the node. According to this ideology, the details of ONBA algorithm are demonstrated as shown in Algorithm 2.

From Algorithm 2 we may know that if every Γ_*v*,*C*_*i*__ is lower than belonging threshold *ξ*, and then the community of node *v* remains unchanged, if there exist the situation that the belonging value of node *v* (denoted Γ_*v*,*C*_*i*__) belongs to community *C*_*i*_ is higher or equal to *ξ*, then delete node *v* from the communities whose community belonging value is lower than *ξ*. After the above operations, the ultimate overlapping community structure is achieved.

It can be seen that in ONBA algorithm when the value of *ξ* is lower, the number of nodes which has tendency is relatively larger, so the overlapping nodes have higher possibility of being adjusted, but the tendency of an overlapping node to a community can easily be satisfied, so there still will be many overlapping nodes in the detected community structure. However, as the value of *ξ* increases, the tendency of a node to a community will be more difficult to be satisfied, which declines the number of the detected overlapping nodes. Of course, when the value of *ξ* is higher, there will be less nodes with tendency, so only a few nodes will be adjusted, thus, the community structure obtained at last will still have a lot of overlapping nodes. Overall, *ξ* plays an essential role in judging a node is overlapping node or not. Therefore, during the analysis of the experiment, the value of *ξ* will be analyzed in detail.

In ONBA algorithm, the time complexity of calculating the community belonging value of every overlapping node is the highest. The time needed in calculating the community belonging value of *L* overlapping nodes is *O*(*kL*), where *k* is the average degree of the nodes in the networks. Since mobile networks are usually of sparse structures, and *k* is constant, the time complexity of ONBA is *O*(*L*). And because *L* is far less than *n* which is the number of nodes in the networks, so the time complexity of ONBA is far lower than *O*(*n* ^2^).

In MRLD algorithm, the time complexity of LLDA is *O*(*m* + *n* ^2^). The running time of the combination phase and the running time of ONBA algorithm are far shorter than *O*(*n* ^2^). Therefore the time complexity of MRLD is *O*(*m* + *n* ^2^).

With the accurate overlapping community structure obtained in the mobile networks, we can perform recommendation to the users of the whole community according to the limited user information.

## 4. Experiments and Evaluation

In order to evaluate the performance of the MRLD algorithm, we use synthetic networks and real-world networks to test and analyze it. Five classic algorithms: CPM [3], LC [4], LFM [11], COPRA [13] and GANET+ [19] are selected to compare with MRLD aiming at proving the validity and the feasibility of the algorithm.

### 4.1. Evaluation Criteria

In order to test the performance of every algorithm, three evaluation criteria are adopted to evaluate the advantages and disadvantages of each algorithm in three aspects: the accuracy of community detection, the accuracy of overlapping nodes detection, and the density of intracommunity connections.

The first criterion is extended normalized mutual information (NMI) [11], it is to test the accuracy of overlapping community detection. Its definition is as follows:

(11) $$\begin{array}{c} \text{NMI}\left(X\;|\;Y\right)=1-\frac{\left[H\left(X\;|\;Y\right)+H\left(Y\;|\;X\right)\right]}{2}, \end{array}$$

where *X* and *Y* are community structures, *H*(*X*∣*Y*) = (1/|*c*′|)∑_*k*_(*H*(*X*_*k*_∣*Y*)/*H*(*X*_*k*_)) represents the normalized condition entropy of a cover *X* with respect to *Y*.

The range for the value of NMI is from 0 to 1; if NMI = 1, it means the detected community structure is completely the same with the true community structure; if NMI = 0, then the detected community structure is entirely different with the true community structure. That is, the value of NMI increases when the accuracy of the detected community structure is improved.

The second criterion is *F*-score [20], which is adopted to evaluate the accuracy of overlapping nodes detection, and its function is defined as follows:

(12) $$\begin{array}{c} F=\frac{2\cdot \text{precision}\cdot \text{recall}}{\text{precision}+\text{recall}}, \end{array}$$

where recall denotes the number of correctly detected overlapping nodes divided by the real number of overlapping nodes, and precision denotes the number of correctly detected overlapping nodes divided by the total number of detected overlapping nodes. The range for *F*-score is from 0 to 1, and the value of *F*-score is higher when the accuracy of overlapping nodes detection is higher.

The third criterion adopted is EQ (see Section 3.2). It is to evaluate the density of intracommunity connections, in which higher EQ values indicate denser intracommunity connections of the community structure.

### 4.2. Synthetic Networks

LFR benchmark networks [21] proposed by Lancichinetti et al. have extremely similar statistical property with real-world networks. So this paper chooses LFR benchmark as the experimental dataset of MRLD. The parameter settings for the LFR benchmark networks are as follows: the network size *N* is set to 200, the average node degree *k* is set to 10, the maximum node degree *k*_max⁡_ is 30, and minimum community size *C*_min⁡_ is 20, the maximum community size *C*_max⁡_ is set to 50, *O*_*n*_ is the number of overlapping nodes, and it is set to either 20 or 100, the number of communities each overlapping node belongs to (denoted *O*_*m*_) is set from 2 to 6, the mixing parameter *γ* varies from 0.1 to 0.4 (the community structure is clearer when the value of *γ* is lower). Because there exist real overlapping community structure in LFR benchmark networks, this paper adopt NMI and *F*-score respectively in this dataset to assess the similarity of the community structure obtained by each algorithm with the true community structure, with the purpose of assessing the detection accuracy of each algorithm.

#### 4.2.1. The Analysis of Belonging Threshold Value *ξ*

**Figure 3:**
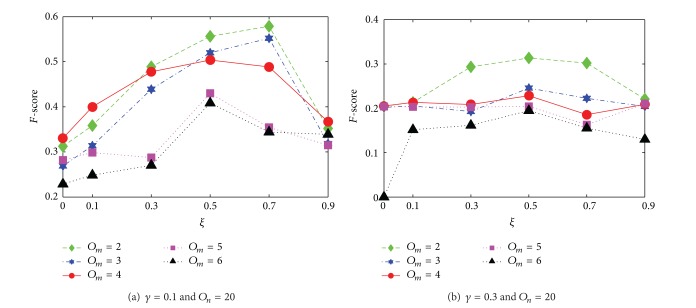
Analysis of threshold value *ξ* of MRLD in terms of *F*-score.

The purpose of this section is to find out which threshold value could ensure the optimal accuracy of MRLD.  Figure 3 shows the comparison of *F*-score in different network structures, when the threshold value *ξ* of MRLD varies from 0 to 0.9. As we can see, when *ξ* = 0.1 − 0.9, the *F*-score values are higher than the corresponding *F*-score values when *ξ* = 0. This indicates that ONBA is effective. Besides, we can also see that if *ξ* = 0.5, only when *γ* = 0.1 and *O*_*m*_ = 2 or 3, the *F*-score values of the detected communities are lower than the corresponding *F*-score values when *ξ* = 0.7. In other cases, when *ξ* = 0.5 all *F*-score reached their optimal values. So in this paper, the threshold value *ξ* of MRLD is set to 0.5.

#### 4.2.2. Comprehensive Analyses of MRLD

**Figure 4:**
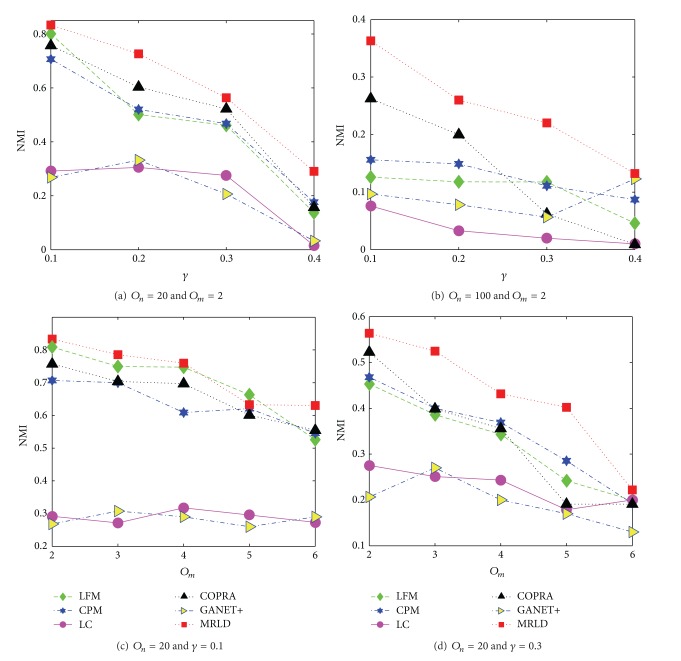
Comparison of MRLD with other algorithms in terms of NMI.

Figure 4 shows the comparison of MRLD with other algorithms in terms of NMI values. As we can see, no matter in the networks with lower overlapping density presented in Figures 4(a), 4(c), and 4(d) or in the networks with higher overlapping density presented in Figure 4(b), the NMI values of MRLD is obviously superior to the other algorithms. Therefore, we can come to the conclusion that MRLD possesses a higher accuracy in detecting overlapping communities.

During process of detecting overlapping nodes with LC and GANET+ algorithms, there exists the phenomenon of excessive overlapping nodes detection, which makes it unable to evaluate accurately the accuracy of the two algorithms in detecting overlapping nodes. Therefore, this paper only analyzes the accuracy of overlapping nodes detection of MRLD and the other three algorithms.

**Figure 5:**
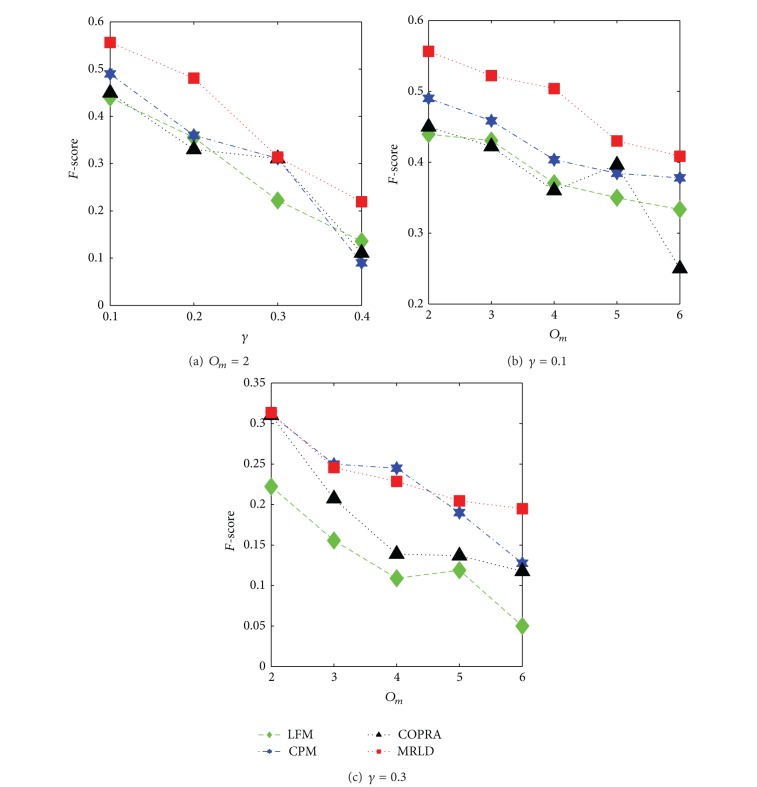
Comparison of MRLD with other algorithms in terms of *F*-score in low-density networks (*O*_*n*_ = 20).

Figure 5 shows the comparison of MRLD with other algorithms in terms of *F*-score in networks with lower overlapping degree. As we can see, only when *γ* = 0.3, *O*_*m*_ = 4, the *F*-score value of the detected communities is lower than the *F*-score of CPM, in other cases, the *F*-score values of MRLD are obviously superior to the *F*-score values of the other algorithms compared. This could prove the high accuracy of MRLD in detecting overlapping nodes.

**Figure 6:**
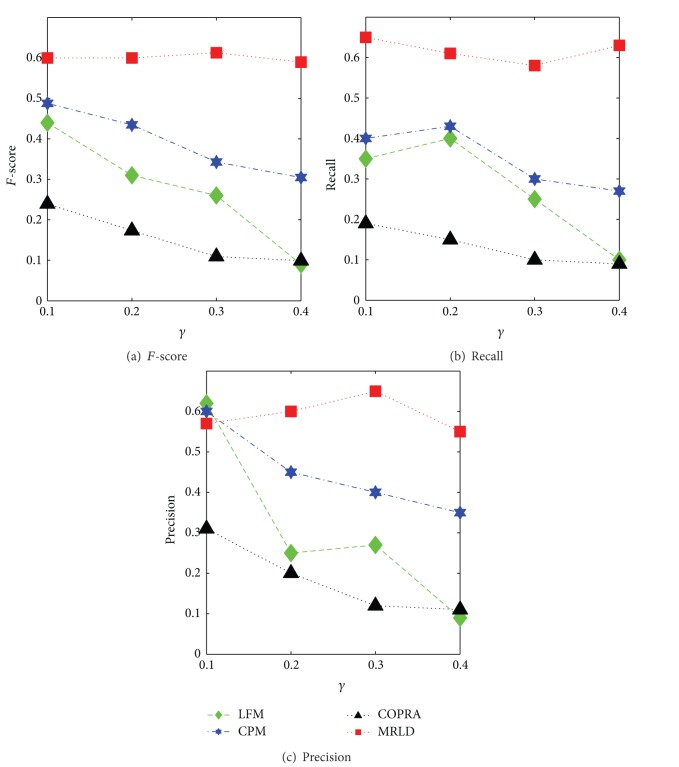
Comparison of MRLD with other algorithms in terms of *F*-score, recall, and precision in high-density networks (*O*_*n*_ = 100, *O*_*m*_ = 2).

Figure 6 shows the comparison of MRLD with other algorithms in terms of recall, precision, and *F*-score values when *O*_*n*_ = 100. The comparison has shown that in networks with higher overlapping density, MRLD could still detect overlapping nodes accurately.

By analyzing NMI, we know that the community structure detected by MRLD is more accurate, and by analyzing *F*-score we can come to the conclusion that MRLD is also more accurate in detecting overlapping nodes. To sum up, MRLD has excellent ability to detect overlapping community structure in networks.

### 4.3. Real-World Networks

#### 4.3.1. Traditional Real-World Networks

As the topological property of real-world networks is different from the topological property of synthetic networks, we use some well-known real-world networks to further evaluate the performance of MRLD.  Table 1 illustrates the datasets of real-world networks used in this paper.

In real-world networks, there are few networks whose community structure is already known, and the networks is usually not overlapped, so it is hard to execute good accuracy analysis. Therefore, this paper adopts EQ as the evaluation criterion to assess the density of intracommunity connections of the communities detected by each algorithm.

Table 2 is the comparison of MRLD with other algorithms in terms of EQ. As we can see, the algorithms are performed in eight real-world networks, although in network Lesmis, Football, and Jazz, MRLD does not obtain the optimal EQ value, in the other five networks, the EQ value of MRLD is obviously superior to the EQ values of the other algorithms. This means that MRLD could uncover community structure with dense intraconnections.

#### 4.3.2. Real-World Mobile Networks

**Figure 7:**
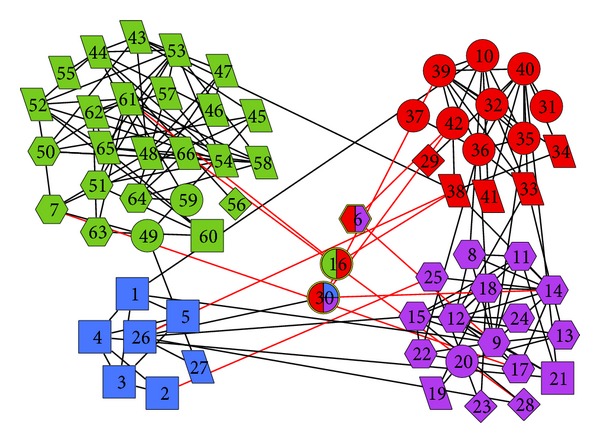
Detection result obtained by MRLD in real-world mobile network.

In order to give a comprehensive display of the performance of MRLD algorithm, we chose 66 students as the study object. All these students are from the same major of a university and they often communicate with the mobile software “Wechat.” The 66 students are presented as nodes in the network and have links between the students who often communicate with each other, 163 links in all. Investigations show that the students are interested in five kinds of news, namely, political news, entertainment news, sports news, military news, and social news.  Figure 7 shows the detection result obtained by MRLD in this network, in which we have five geometric figures: parallelogram, circle, rectangle, hexagon, and rhombus, representing the favorite kind of news of a student in the order of entertainment news, sports news, military news, social news and political news, respectively. As we can see, the network is divided into four communities by MRLD algorithm, and in each community most students have the same favorite news. That is to say, the users in the same densely connected community are more likely to have the same interest. It also proved that in mobile networks, MRLD could aggregate users with the same interest into the same community, that is, it is feasible to perform group recommendation in mobile networks using community as the unit.

## 5. Conclusion and Future Work

In consideration of their privacy and security, mobile users are reluctant to provide precise and complete information to the mobile recommendation systems, which influenced the accuracy of the systems greatly. Focusing on this problem, we propose MRLD algorithm. MRLD first initializes the label of every link to node label which possesses the higher degree when connected by the link, then get the links according to the degree of the nodes (the nodes are connected by the links) in descending order and execute triangle label diffusion to aggregate the links with the same label to one community, thus, to form a dense original community structure. Next, it combines the obtained communities with maximal EQ of greedy search to obtain the optimal overlapping community structure. Afterwards, overlapping nodes belonging analysis (ONBA) is adopted to adjust the overlapping nodes in order to avoid “excessive overlapping” which had appeared in link community detection. On the basis of detecting the overlapping communities precisely, MRLD could improve the accuracy of recommendation by performing targeted recommendation to every community in mobile networks. MRLD algorithm has been tested both on synthetic and real-world networks, and the evaluation results have shown the validity and feasibility of MRLD.

Currently, utilizing community detection technology to perform mobile recommendation has not been studied much. Thus, in the future, we will explore new approaches of mobile recommendation on the basis of community detection to improve the speed and accuracy of mobile recommendation.

## Figures and Tables

**Algorithm 1:**
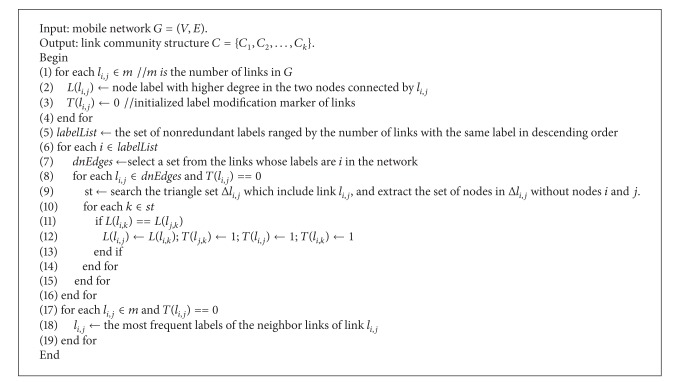
Link label diffusion algorithm (LLDA).

**Algorithm 2:**
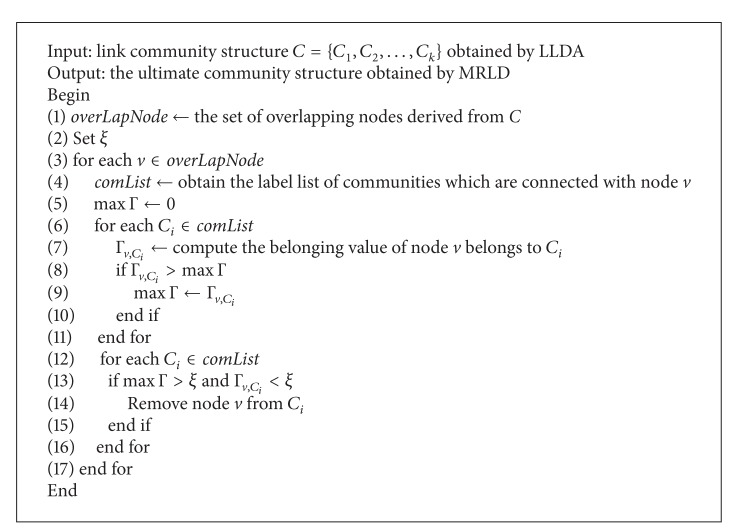
Overlapping nodes belonging analysis (ONBA).

**Table 1:** Datasets of real-world networks.

Network	Nodes	Links	Description
Karate	34	78	Zachary's karate club [22]
Dolphins	62	159	Dolphin social network [23]
Lesmis	77	254	Novel *Les Misérables* [24]
Polbooks	105	441	Books about US politics [25]
Football	115	613	American college football [26]
Jazz	198	2742	Jazz musicians network [27]
Polblogs	1224	19022	Political blogs [28]
Email	1133	5451	Emails of human interactions [29]

**Table 2:** Comparison of MRLD with other algorithms in terms of EQ.

EQ	MRLD	CFINDER	LFM	COPRA	LC	GANET+
Karate	**0.3717**	0.1072	0.2146	0.3239	0.1220	0.081
Dolphins	**0.5127**	0.2885	0.2374	0.4206	0.1514	0.1388
Lesmis	0.4764	0.1855	**0.4812**	0.4779	0.2484	0.1402
Polbooks	**0.4833**	0.4304	0.3476	0.4586	0.2198	0.0946
Football	0.5135	0.5593	0.5098	**0.6008**	0.0754	0.1196
Jazz	0.1573	0.0043	0.2334	**0.3428**	0.0281	0.034
Polblogs	**0.3115**	/	0.1476	0.3035	0.0012	0.027
Email	**0.4217**	0.2641	0.1822	0.3523	0.0261	0.1121

## References

[1] Wang LC, Meng XW, Zhang YJ (2011). A cognitive psychology-based approach to user preferences elicitation for mobile network services. *Acta Electronica Sinica*.

[2] Newman MEJ, Girvan M (2004). Finding and evaluating community structure in networks. *Physical Review E*.

[3] Palla G, Derenyi I, Farkas I, Vicsek T (2005). Uncovering the overlapping community structure of complex networks in nature and society. *Nature*.

[4] Ahn YY, Bagrow JP, Lehmann S (2010). Link communities reveal multi-scale complexity in networks. *Nature*.

[5] Shen HW, Cheng XQ, Cai K, Hu MB (2009). Detect overlapping and hierarchical community structure in networks. *physical A: Statistical Mechanics and Its Applications*.

[6] Groh G, Ehmig C Recommendations in taste related domains: collaborative filtering vs. social filtering.

[7] Ma H, King I, Lyu MR Learning to recommend with social trust ensemble.

[8] Huang WH, Meng XW, Wang LC (2011). A collaborative filtering algorithm based on users’ social relationship mining in mobile communication network. *Journal of Electronics & Information Technology*.

[9] Quercia D, Capra L FriendSensing: recommending friends using mobile phones.

[10] Girvan M, Newman MEJ (2002). Community structure in social and biological networks. *Proceedings of the National Academy of Sciences of the United States of America*.

[11] Lancichinetti A, Fortunato S, Kertész J (2009). Detecting the overlapping and hierarchical community structure in complex networks. *New Journal of Physics*.

[12] Lancichinetti A, Radicchi F, Ramasco JJ, Fortunato S (2011). Finding statistically significant communities in networks. *PLoS ONE*.

[13] Gregory S (2010). Finding overlapping communities in networks by label propagation. *New Journal of Physics*.

[14] Wu ZH, Lin YF, Gregory S, Wan HY, Tian SF (2012). Balanced multi-label propagation for overlapping community detection in social networks. *Journal of Computer Science and Technology*.

[15] Xie JR, Szymanski BK, Liu X Slpa: uncovering overlapping communities in social networks via a speaker-listener interaction dynamic process.

[16] Evans TS, Lambiotte R (2009). Line graphs, link partitions, and overlapping communities. *Physical Review E*.

[17] Kim Y, Jeong H (2011). Map equation for link community. *Physical Review E*.

[18] Bagrow JP, Bollt EM (2005). Local method for detecting communities. *Physical Review E*.

[19] Pizzuti C Overlapped community detection in complex networks.

[20] Xie JR, Kelley S, Szymanski BK (2013). Overlapping community detection in networks: the state of the art and comparative study. *ACM Computing Surveys*.

[21] Lancichinetti A, Fortunato S, Radicchi F (2008). Benchmark graphs for testing community detection algorithms. *Physical Review E*.

[22] Zachary WW (1977). An information flow model for conflict and fission in small groups. *Journal of Anthropological Research*.

[23] Lusseau D (2003). The emergent properties of a dolphin social network. *Proceedings of the Royal Society B: Biological Sciences*.

[24] Knuth DE The Stanford GraphBase: A Platform for Combinatorial Computing. http://www-cs-faculty.stanford.edu/~uno/sgb.html.

[25] Newman MEJ (2006). Modularity and community structure in networks. *Proceedings of the National Academy of Sciences of the United States of America*.

[26] Girvan M, Newman MEJ (2002). Community structure in social and biological networks. *Proceedings of the National Academy of Sciences of the United States of America*.

[27] Gleiser P, Danon L (2003). Community structure in jazz. *Advances in Complex Systems*.

[28] Adamic LA, N.Glance The political blogosphere and the 2004 US election: divided they blog.

[29] Guimerà R, Danon L, Díaz-Guilera A, Giralt F, Arenas A (2003). Self-similar community structure in a network of human interactions. *Physical Review E*.

